# Serum N-Glycome Diversity in Teleost and Chondrostrean Fishes

**DOI:** 10.3389/fmolb.2021.778383

**Published:** 2021-11-10

**Authors:** Kazuhiro Aoki, Tadahiro Kumagai, René Ranzinger, Carl Bergmann, Alvin Camus, Michael Tiemeyer

**Affiliations:** ^1^ Complex Carbohydrate Research Center, University of Georgia, Athens, GA, United States; ^2^ Procter & Gamble, Takasaki, Japan; ^3^ College of Veterinary Medicine, University of Georgia, Athens, GA, United States

**Keywords:** glycomics, mass spectrometry, fish, serum, N-glycan

## Abstract

Recent advances in carbohydrate chemistry, chemical biology, and mass spectrometric techniques have opened the door to rapid progress in uncovering the function and diversity of glycan structures associated with human health and disease. These strategies can be equally well applied to advance non-human health care research. To date, the glycomes of only a handful of non-human, non-domesticated vertebrates have been analyzed in depth due to the logistic complications associated with obtaining or handling wild-caught or farm-raised specimens. In contrast, the last 2 decades have seen advances in proteomics, glycoproteomics, and glycomics that have significantly advanced efforts to identify human serum/plasma biomarkers for various diseases. In this study, we investigated N-glycan structural diversity in serum harvested from five cultured fish species. This biofluid is a useful starting point for glycomic analysis because it is rich in glycoproteins, can be acquired in a sustainable fashion, and its contents reflect dynamic physiologic changes in the organism. Sera acquired from two chondrostrean fish species, the Atlantic sturgeon and shortnose sturgeon, and three teleost fish species, the Atlantic salmon, Arctic char, and channel catfish, were delipidated by organic extraction and the resulting protein-rich preparations sequentially treated with trypsin and PNGaseF to generate released N-glycans for structural analysis. Released N-glycans were analyzed as their native or permethylated forms by nanospray ionization mass spectrometry in negative or positive mode. While the basic biosynthetic pathway that initiates the production of glycoprotein glycan core structures is well-conserved across the teleost fish species examined in this study, species-specific structural differences were detected across the five organisms in terms of their monosaccharide composition, sialylation pattern, fucosylation, and degree of *O*-acetylation. Our methods and results provide new contributions to a growing library of datasets describing fish N-glycomes that can eventually establish species-normative baselines for assessing N-glycosylation dynamics associated with pathogen invasion, environmental stress, and fish immunologic responses.

## Introduction

Fish have been a vital source of protein and other nutrients throughout human history. On a global scale, 50% of the fish consumed are currently produced by aquaculture; therefore, understanding fish immunology in relation to stress and disease is crucial for sustaining commercial production (Torrissen et al., 2011). Complex carbohydrates, called glycan moieties, are linked to lipids and proteins, forming a major component of the surface of normal and transformed cells. These glycans have been shown to modulate interactions between cells, between cells and their tissue micro-environment, as well as to mediate immune cell and growth factor signaling (Ohtsubo and Marth, 2006; Moremen et al., 2012). Pronounced changes in the structure of the glycans attached to lipids and proteins have been reported in tissue development and disease progression in humans as well as model organisms such as zebrafish, fruit flies, and mice (Wang et al., 2001; Nakakita et al., 2005; Ten Hagen et al., 2009; Baas et al., 2011; Chu et al., 2013; Hanzawa et al., 2017; Frappaolo et al., 2018; Cervoni et al., 2020; Rehan et al., 2020).

While much effort has been expended over the past half-century toward characterizing the glycomes of humans and in-bred animal model systems, little experimental effort has been applied toward understanding glycomic diversity across fish phylogeny. Additionally, relatively little is known about the environmental, genetic, and molecular mechanisms that regulate the expression of species-, tissue-, and cell-specific glycans in any context in fish or other organisms, despite the ample evidence that glycans play essential roles in host/microbe interactions at multiple levels (Rostand and Esko, 1997). Furthermore, exposure to microorganisms induces host responses that may include altered glycan expression. Thus, understanding the diversity and dynamics of glycan expression in fish populations should provide important baseline constraints for assessing the health of cultured and wild-caught fish as well as for tracking aquaculture productivity and product quality.

Several previous studies have investigated the structural diversity of fish O-linked and N-linked glycoprotein glycans. Glycans have been harvested from gill, skin, intestinal mucins, and eggs of fish in the family *Salmonida*e (Kanamori et al., 1990; Iwasaki et al., 1992; Jin et al., 2015; Venkatakrishnan et al., 2019; Benktander et al., 2020). In Atlantic salmon (*Salmo salar*) skin, O-glycans contain sialylated structures composed of a NeuAc-NeuAc (di-SA) signature glycan motif that has also been detected on N-glycans of this specie’s cysteine proteinase inhibitors (Ylonen et al., 2001). In addition, N-glycans with di-SA motifs have also been detected in rainbow trout (*Oncorhynchus mykiss*) ovarian fluid (Funakoshi et al., 1997). Glycomic studies on the gastrointestinal (GI) tracts of Atlantic salmon, Arctic char (*Salvelinus alpinus*), and carp species (genus *Cyprinus*) demonstrated that the repertoire of mucin O-glycan structures are different from those found in other organs (Venkatakrishnan et al., 2019). As expected from other animal studies, these glycan profiles are not static; they respond to stress and inflammatory insults. O-Glycan analysis of intestinal mucin from Arctic char fed with full-fat ground soy beans (FFSB) to induce inflammation demonstrated shifts in glycan profiles (Venkatakrishnan et al., 2019). Among variations of glycan structures associated with the fish immune response, altered modification of sialic acid is specifically involved in stress-inducible responses. It has been reported that desialylation of intestinal and skin mucins purified from Atlantic salmon results in reduction of *Aeromonas salmonicida* binding to the mucins and increases growth of the pathogen (Jin et al., 2015). These differences suggest that glycomic change is closely associated with immune responses to pathogen invasion. Bacterial infection triggers the immune system and alters the glycosylation pattern of immune cells and the immunoglobulins they produce.

In this study, blood samples were acquired from five fish species that are relevant for commercial aquaculture in order to profile their serum N-glycans. The results provide a landscape of the range of structural diversity associated with unchallenged fish, identify novel structural features, and lay the foundation for future perturbation or longitudinal health surveys. The species chosen for analysis also allowed us to investigate the species-related glycan diversity by comparing the N-glycans of teleost and chondrostean fishes.

## Materials and Methods

### Fish Blood Samples

Whole blood was collected from laboratory or pond reared Atlantic salmon, Arctic char, channel catfish (*Ictalurus punctatus*), Atlantic sturgeon (*Acipencer oxyrinchus*), and shortnose sturgeon (*Acipenser brevirostrum*) as described below. Prior to collection, fish were anesthetized in 100–300 mg/L tricaine methane sulfonate (MS-222) (Argent Chemical Laboratories, Redmond, WA, United States). Depending on fish size, 0.5–5 ml of blood was collected by puncture of the caudal vein using a 24–21 g needle (from smaller to larger fish) and syringe. To avoid hemolysis, needles were removed from syringes and the blood transferred immediately to a red top (clot) vacutainer tube (Becton Dickinson, Franklin Lakes, NJ, United States) and placed on ice. Clotted blood samples were briefly centrifuged and the serum removed from the precipitated red blood cells before freezing at −80°C until used. Approximately 10–20 μL of serum was used for glycan analysis.

## Materials

PNGaseF (N-glycanase) was obtained from Prozyme (San Leandro, CA). Sodium hydroxide (50%) was purchased from Fisher Scientific. Sep-Pak C18 disposable columns were obtained from Waters Corporation (Milford, MA, United States). Oligosaccharide kit for reference standards, trypsin, bovine pancreas ribonuclease B (RNaseB), α-galactosidase from green coffee beans, β-galactosidase from *Aspergillus oryzae*, and all other reagents were purchased from Sigma-Aldrich (St Louis, MO, United States).

### Release of N-Glycan From Serum Glycoprotein

Serum samples were delipidated prior to N-glycan preparation as described previously (Aoki et al., 2007; Boccuto et al., 2014; Mehta et al., 2016; Rosenbalm et al., 2020). For N-glycan analysis, 0.5 mg of protein-rich powder was resuspended in 200 μL of trypsin buffer (0.1 M Tris-HCl, pH 8.2, containing 1 mM CaCl_2_) by sonication and boiled for 5 min. After cooling to room temperature, 25 μL of trypsin solution (2 mg/ml in trypsin buffer) and 25 μL of chymotrypsin solution (2 mg/ml in trypsin buffer) were added. Digestion was allowed to proceed for 18 h at 37°C before the mixture was boiled for 5 min. Insoluble material was removed by centrifugation and the supernatant was removed and dried by vacuum centrifugation. The dried peptide and glycopeptide mixture was resuspended in 250 μL of 5% acetic acid (v/v) and loaded onto a Sep-Pak C18 cartridge column. The cartridge was washed with 10 column volumes of 5% acetic acid. Glycopeptides were eluted stepwise, first with 3 volumes of 20% isopropyl alcohol in 5% acetic acid and then with 3 volumes of 40% isopropyl alcohol in 5% acetic acid. The 20 and 40% isopropyl alcohol steps were pooled and evaporated to dryness. Dried glycopeptides were resuspended in 50 μL of 50 mM sodium phosphate buffer, pH 7.5, for digestion with PNGaseF. Following PNGase digestion for 18 h at 37°C, released N-linked glycans were separated from peptides and enzyme by passage through a Sep-Pak C18 cartridge. The digestion mixture was reconstituted in 200 μL of 5% acetic acid and loaded onto a Sep-Pak C18 cartridge column. The column pass-through and an additional elution with 3 volumes of 5% acetic acid, containing released N-glycans, were collected together and evaporated to dryness.

### Exoglycosidase Digestions

α-Galactosidase from green coffee beans and β-galactosidase from *Aspergillus oryzae* were used to cleave terminal galactose residues from oligosaccharides (Miller et al., 2018). For exoglycosidase treatments, a total of 0.4–0.6 units of enzyme were used to cleave terminal galactose residues as follows. For α-galactosidase treatment, N-glycopeptides were digested in 50 µL of 100 mM citrate-phosphate buffer, pH 6.5 at 25°C in the presence of γ-galactonolactone (0.5 mg/ml), an inhibitor of β-galactosidase. For complete digestion of terminal α-galactosyl residues, the N-glycopeptides were treated with 0.2 units of α-galactosidase for 24 h, then a second 0.2 unit aliquot of the enzyme was added and incubated for an additional 18 h. For β-galactosidase treatment, N-glycopeptides treated with α-galactosidase were desalted on a Sep-Pak C18 cartridge prior to digestion. Purified de-α-galactosyl glycopeptides were digested in 50 µL of 20 mM citrate-phosphate buffer, pH 4.5 at 30°C. To remove terminal β-galactosyl residues, the N-glycopeptides were first treated with 0.3 units of β-galactosidase for 24 h, then a second 0.3 unit of the enzyme was added and incubated for an additional 18hrs. The reaction mixtures were desalted on a Sep-Pak C18 cartridge prior to N-glycan release.

### Analysis of N-Glycan by Mass Spectrometry

Released N-glycans were analyzed as their permethylated or native forms by nanospray ionization mass spectrometry (NSI-MS) in positive and negative ion mode. For MS of permethylated form N-glycans in positive ion mode, permethylated N-glycans were dissolved in 50 µL of 1 mM sodium hydroxide in methanol/water (1:1) for infusion into a linear ion trap mass spectrometer (Orbi-LTQ; ThermoFisher Scientific) using a nanospray source at a syringe flow rate of 0.60 μL/min and capillary temperature set to 210°C (Aoki et al., 2007; Boccuto et al., 2014; Mehta et al., 2016). To optimize data acquisition, the MS method was tuned using a mixture of permethylated N-glycans prepared from ribonuclease B glycoprotein. For MS of native form N-glycans, native N-glycans were reconstituted in 50 μL of methanol/2-propanol/1-propanol/13 mM aqueous ammonium acetate (16:3:3:2 by volume) for infusion and analyzed in negative ion mode (Schnaar et al., 2008; Kumagai et al., 2013). The instrument parameters for negative ion mode were optimized utilizing native sulfated Lewis A trisaccharide. For fragmentation by collision-induced dissociation (CID) in MS/MS and MSn, a normalized collision energy of 35–40% was used. Detection and relative quantification of the prevalence of individual N-glycans was accomplished using the total ion mapping (TIM) functionality of the Xcalibur software package version 2.0 (ThermoFisher Scientific) as previously described (Aoki et al., 2007; Boccuto et al., 2014; Mehta et al., 2016). For TIM, the m/z range from 600 to 2000 was automatically scanned in successive 2.8 mass unit windows with a window-to-window overlap of 0.8 mass units, which allowed the naturally occurring isotopes of each N-glycan species to be summed into a single response, thereby increasing detection sensitivity. Most N-glycan components were identified as singly, doubly, or triply charged, sodiated species (M + Na) in positive mode. Peaks for all charge states were summed for quantification. For quantification of permethylated N-glycans, permethylated maltotetraose (Dp4, observed at m/z 899) was co-injected with 5% of permethylated N-glycans (N-glycans from 0.5 μL of fish serum) as an external standard (Mehta et al., 2016). Graphic representations of N-glycan monosaccharide residues are consistent with the Symbol Nomenclature for Glycans (SNFG) as adopted by the glycomics and glycobiology communities [21]. The angle at which branching monosaccharide residues are depicted in graphic representations is not meant to infer specific linkage positions or anomeric configuration, some of which remain to be determined by future analysis. Glycomics data and metadata were obtained and are presented in accordance with MIRAGE standards and the Athens Guidelines (York et al., 2014; Liu et al., 2017). All raw mass spectrometric data was deposited at GlycoPost, accession #GPST000210 (Watanabe et al., 2021).

## Results

### N-Glycan Profiles of Salmonid Sera

Three replicates of sera harvested from separate fish (biological replicates) were subjected to N-glycan profiling and demonstrated high reproducibility (Supplementary Figures 5A,B). Typically, 10 mg of protein-rich powder was obtained from 200 μL of fish sera. Serum N-glycans were released from 0.5 mg of serum glycoproteins by PNGaseF and analyzed by NSI-MS as their permethylated derivative (PerMe) or underivatized (native). The conditions of the permethylation reaction remove base-labile modifications such as *O*-acetylation; analysis of native glycans allowed the detection of these modifications. For quantification, the threshold was set at 3% of the most intense peak detected on MS1 for each sample. N-glycan structural assignments were confirmed by MSn fragmentation.

**FIGURE 1:**
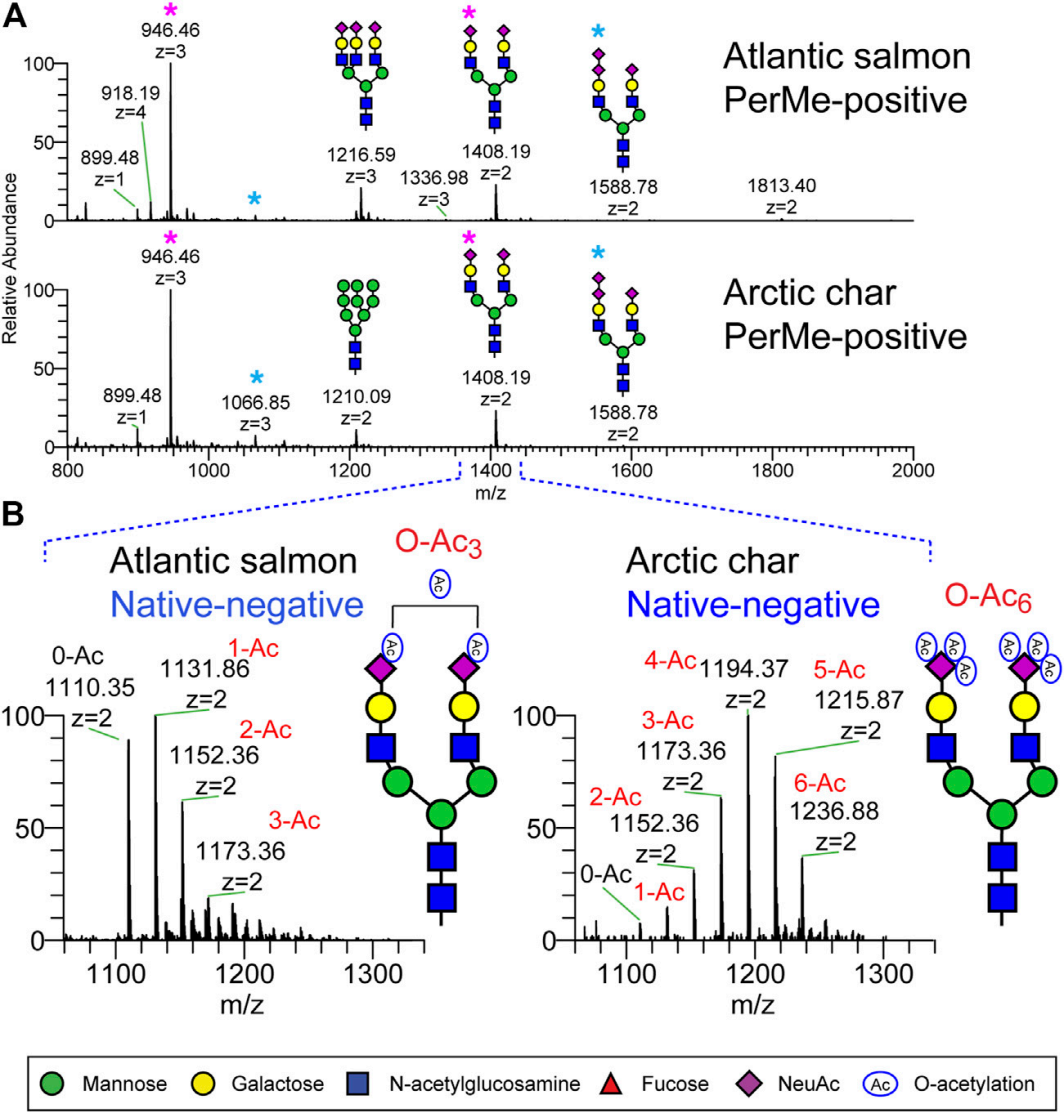
N-Glycans detected in serum of Atlantic salmon and arctic char by NSI-MS. N-Glycans released from fish sera glycoproteins by PNGaseF were analyzed as their permethylated or non-permethylated (native) forms. **(A)** Full MS spectra of permethylated N-glycans analyzed in positive ion mode. Both salmon species express a bi-antennary disialylated N-glycan observed at m/z 1408 (2+) and 946 (3+) as the most abundant N-glycan component (pink asterisk). The bi-antennary trisialylated N-glycan carrying NeuAc-NeuAc disaccharide, observed at m/z 1588.78 (2+) and 1066.85 (blue asterisk), are also detected in both species. Permethylated maltotetrasaccharide (Dp4) was added as internal standard for quantification and is observed at m/z 899.48. **(B)** Native N-glycans were analyzed in negative ion mode. Expanded view of a subregion of the full MS highlights the presence of *O*-acetylated sialic acid on bi-antennary disialylated N-glycan in the *Salmonidae* species.

Representative permethylated serum N-glycan profiles of Atlantic salmon and Arctic char, both of which belong to the family *Salmonidae*, are shown in Figure 1. The N-glycan of highest prevalence in both species was observed at m/z 1408.19 (2+)/946.46 (3+) and corresponds to a bi-antennary disialylated N-glycan widely expressed in many animal species including humans, mice, swine, and zebrafish (Stumpo and Reinhold, 2010; Kadirvel et al., 2012; Yamakawa et al., 2018). Both Atlantic salmon and Arctic char express bi-antennary trisialylated N-glycan carrying NeuAc-NeuAc disaccharide (di-SA) observed at m/z 1588.78. The disialylated glycan motif has been previously identified in both salmonid species (Ylonen et al., 2001; Jin et al., 2015; Venkatakrishnan et al., 2019). The tri-antennary trisialylated N-glycan at m/z 1216.59 (3+) is abundant in the Atlantic salmon but essentially undetected in Arctic char in the MS1 spectra. Both fish species express low amounts of high mannose type N-glycans. The most complex N-glycan is a tri-antennary tetrasialylated N-glycan detected at m/z 1336.98 (3+) with di-SA in both Atlantic salmon and Arctic char although the relative abundance of this structure was very low (0.38 and 0.12%, respectively). The representative MS2 spectra used to characterize the structure of N-glycans with di-SA observed at m/z 1066 (2+) and 1336 (3+) are shown in Supplementary Figure S1. The di-SA motif is found on 5% of the total N-glycan pool in Arctic char only 2% of the total in Atlantic salmon (Figure 7).

The structures of the major N-glycans were similar in the two salmonid species when analyzed as their permethylated forms in positive ion mode (Figure 1A). However, the degree of *O*-acetylation of sialic acid was significantly different when the native forms of N-glycans (non-permethylated) were analyzed in negative ion mode (Figure 1B). The most abundant *O*-acetylated N-glycans in Atlantic salmon are bi-antennary disialylated N-glycans in which the sialic acid residues were modified with between zero and three *O*-acetyl groups. In contrast, the sialic acids in Arctic char were modified with as many as six *O*-acetyl groups. The detected ions and their compositions are summarized in Supplementary Tables 1A,B. A high level of glycan sialylation was common to both species, although the Atlantic salmon had a greater relative abundance of the trisialylated N-glycan.

### N-Glycan Profiles of Sturgeon Sera

**FIGURE 2:**
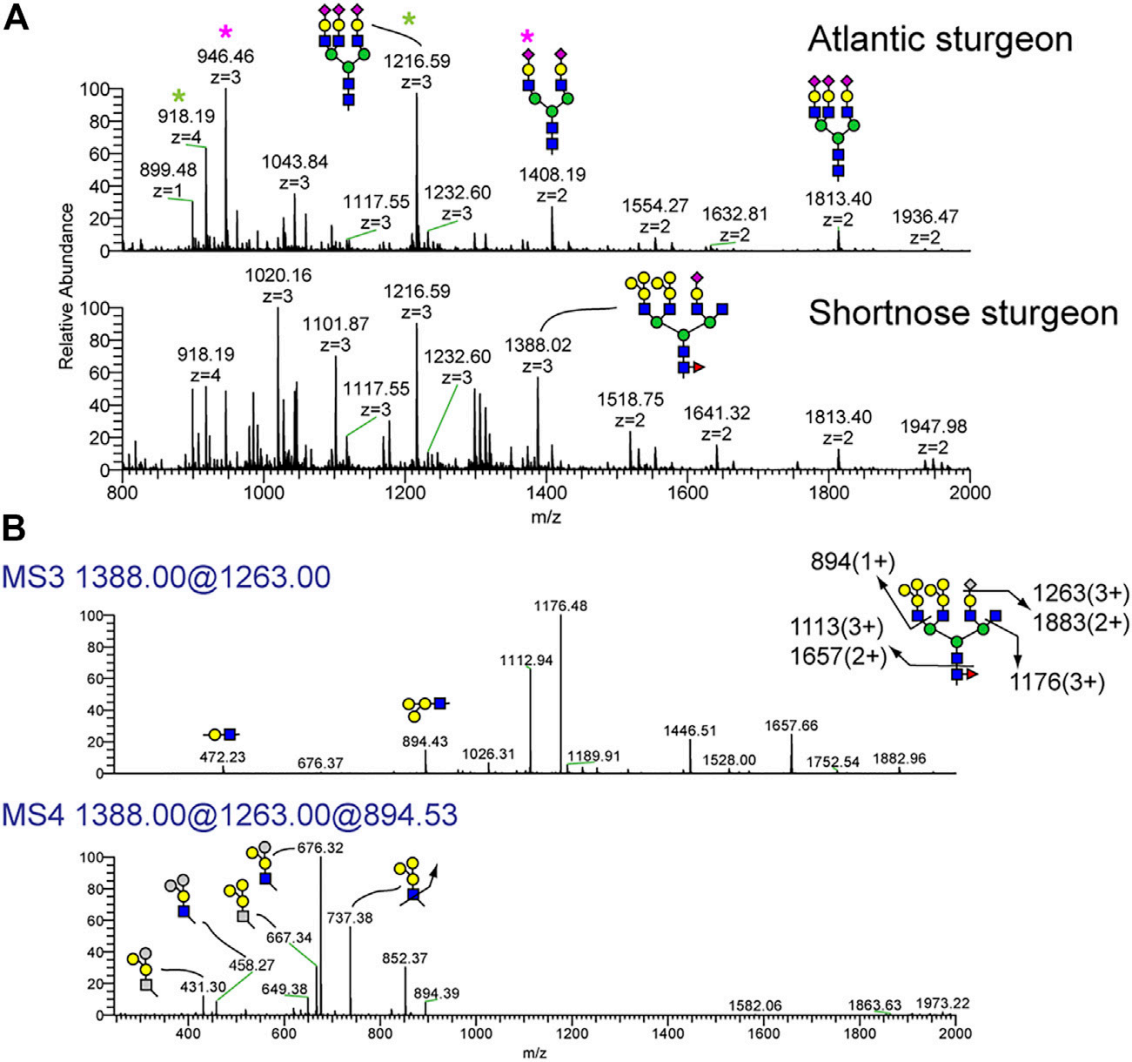
N-Glycans detected in serum of Atlantic sturgeon and shortnose sturgeon by NSI-MSn. Permethylated N-glycans were analyzed on NSI-MS. **(A)** The major N-glycans in Atlantic sturgeon were detected as bi-antennary disialylated (pink asterisk) and tri-antennary trisialylated structures (green asterisk). Shortnose sturgeon serum glycoproteins also carry the same N-glycan structures in addition to more complex, terminally branched glycans. **(B)** NSI-MSn fragmentation of the Shortnose sturgeon N-glycan detected at m/z = 1388 (3+). MS3 of the permethylated N-glycan with composition of NeuAc1Hex7HexNAc4M3N2F1 generates a fragment ion at m/z = 894 corresponding to Hex3HexNAx1. MS4 fragmentation of the signature ion at m/z = 894 generates fragments indicating that the hexose units are configured in a branch, not linear form.

N-glycan profiles of three biological replicates of Atlantic and shortnose sturgeon sera are shown in Supplementary Figures 5C,D, again demonstrating the reproducibility of the analytic approach. The permethylated forms of N-glycans from representative serum samples of Atlantic sturgeon and shortnose sturgeon are shown in Figure 2. We observed significant differences in N-glycan components between the two sturgeon species (Figure 2A). Similar to Atlantic salmon and Arctic char, both sturgeon species express bi-antennary disialylated and tri-antennary trisialylated N-glycans, detected at m/z 1408.19 (2+) and 1813.40 (2+), as their major sialylated N-glycans. However, the shortnose sturgeon possesses N-glycans capped with its own characteristic motif. The MS profile of the permethylated shortnose sturgeon sera glycans demonstrated the presence of N-glycan components detected at m/z 1020.16 (3+), 1101.87 (3+) and 1388.02 (3+). The ion detected at m/z 1388.02 (3+) corresponds to tetra-antennary N-glycans with monosaccharide composition of NeuAc1deoxyHex1Hex10HexNAc6. The high abundance of Hex residues in this composition does not match conventional tetraantennary templates. Moreover, shortnose sturgeon exhibited abundant fucosylation on the chitobiose core (core fucosylation), a minor feature of the Atlantic sturgeon profile.

To further characterize the structural feature associated with the high Hex abundance in shortnose sturgeon, sequential MSn analysis was carried out on the ion detected at m/z 1388.02 (Figure 2B). MS3 spectra for m/z 1388.02 was obtained from a fragment ion produced from neutral loss of a terminal single sialic acid residue, m/z 1263.00. This MS3 data indicates that sialic acid is attached to the terminal galactose of a LacNAc disaccharide, consistent with the NeuAc-Gal-GlcNAc motif broadly observed in animal species. However, we observed a unique fragment ion in the MS4 spectra obtained by fragmentation of the MS3 ion at m/z 894.4, demonstrating the presence of a novel glycan epitope composed of Hex3HexNAc1 with a branched tri-hexose motif of Hex1(Hex1)Hex1HexNAc1.

**FIGURE 3:**
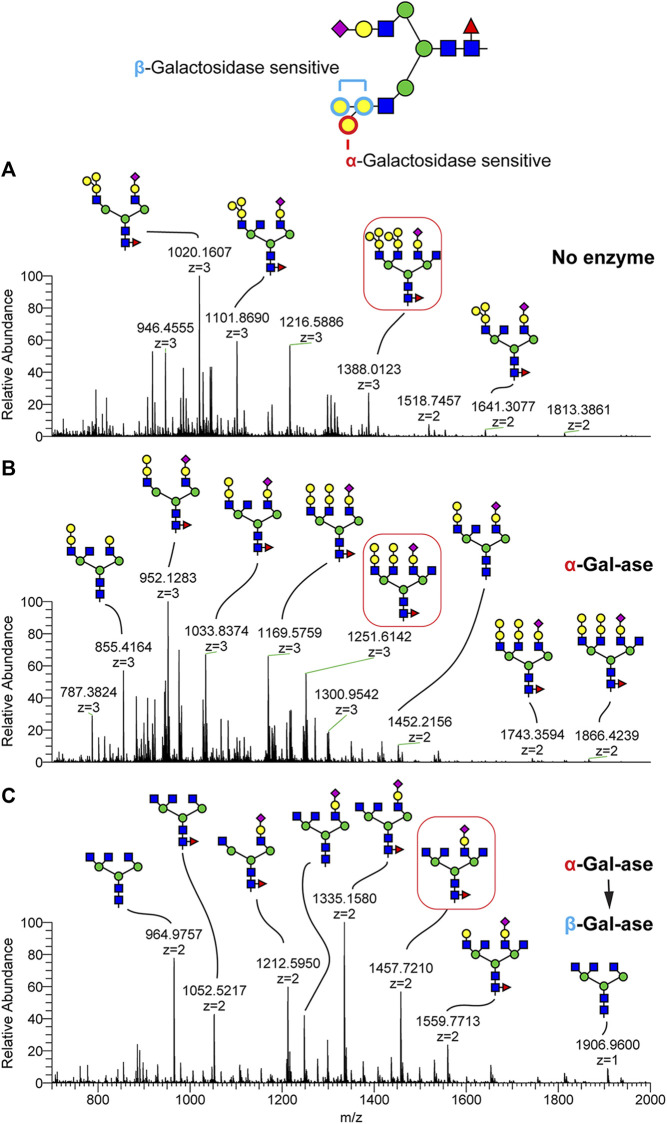
Validation of the shortnose sturgeon terminal glycan motif by exoglyosidase treatment. Glycopeptides harvested from shortnose sturgeon serum were treated with α and β-galactosidases and the N-Glycans were released by PNGaseF and analyzed as their permethylated derivatives by NSI-MS/MS. **(A)** N-glycan profile without enzyme digestion. **(B)** N-glycan profile following α-galactosidase digestion. **(C)** N-glycan profile following sequential α-galactosidase then β-galactosidase digestion. The glycan motif composed of Hex (Hex)HexHexNAc tetrasaccharide (e.g., boxed N-glycan at m/z 1388) is digested to Hex-Hex-HexNAc by β-galactosidase **(B)**. The glycan motif composed of Hex-Hex-HexNAc is digested to HexNAc by sequential treatment of β-galactosidase following α-galactosidase treatment.

To assess the linkage type and topology of this tetrasaccharide glycan epitope, we digested the glycan mixture with α- and β-galactosidases (Figure 3). We also utilized in-source fragmentation (ISF) to generate structurally informative MS1 ions. By applying 100% of the ISF energy, N-glycan structures produced signature fragment ions corresponding to terminal glycan epitopes which were efficiently detected at MS1 without triggering MSn fragmentation. As shown in Supplementary Figure S2, the tetrasaccharide epitope observed at m/z 894 in the N-glycan control was completely shifted to m/z 690 by treatment of α-galactosidase, indicating that one of the terminal Hex is an α-linked galactose residue attached on the Hex1-Hex1-HexNAc trisaccharide. This trisaccharide terminal structure was also detected in MS spectra without ISF following α-galactosidase treatment (Figure 3B). We further treated N-glycan with β-galactosidase following α-galactosidase treatment and detected complete loss of the terminal Hex motif, demonstrating that the sequence of the tetrasaccharide is Galα(Galβ)GalβHexNAc. For example, N-glycan in shortnose sturgeon detected at m/z 1388.02 (3+) with composition of NeuAc1deoxyHex1Hex10HexNAc6 is shifted to m/z 1251.61 (3+)/1866.42 (2+) with composition of NeuAc1deoxyHex1Hex8HexNAc6 by α-galactosidase treatment. By sequential treatment of α- and β-galactosidases, three Hex residues were completely cleaved and produced the molecular ion at m/z 1457.72 (2+), demonstrating that the tetrasaccharide of Galα(Galβ)GalβHexNAc is converted to HexNAc. We also noticed that a few highly complex N-glycan structures, such as the ion detected at m/z 1559.77 (2+), were only partially digested by the galactosidases, suggesting steric interference based on high glycan complexity (Figure 3C). We also applied the ISF method and exoglycosidase treatment to the Atlantic sturgeon N-glycans and determined that both sturgeon species carry the same glycan epitope composed of Galα(Galβ)GalβHexNAc but differ in its relative abundance (Supplementary Figure S3).

We evaluated the non-permethylated (native) forms of the Atlantic sturgeon glycans because this species expressed a high abundance of di- and tri-sialylated N-glycans compared to the shortnose species. We detected sialic acid residues with between zero and one *O*-acetyl modification (Supplementary Figure S4). The observed ions and their glycan compositions are summarized in Supplementary Tables S1C,D.

### N-Glycan Profile of Channel Catfish Serum

**FIGURE 4:**
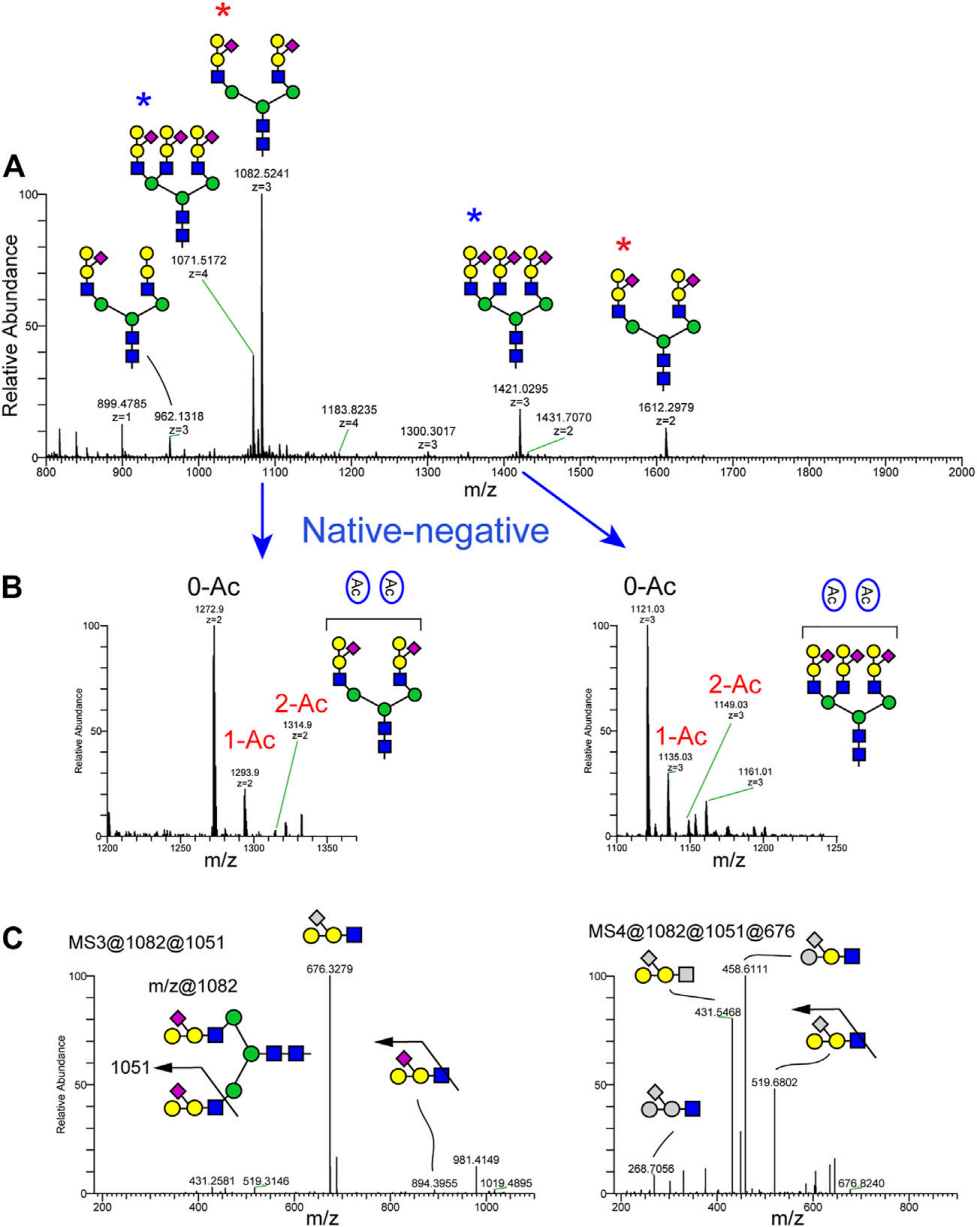
N-Glycans detected in serum of channel catfish by NSI-MSn. N-Glycans released from fish sera glycoproteins by PNGaseF were analyzed as their permethylated or non-permethylated (native) forms. **(A)** Full MS spectra of permethylated N-glycans analyzed in positive ion mode demonstrate that the most abundant N-glycan is a bi-antennary disialylated N-glycan detected at m/z 1082 (3+) and 1612 (2+) (red asterisk). The second-most abundant N-glycan is a tri-antennary trisialylated N-glycan observed at m/z 1071 (2+) and 1421 (blue asterisk). Permethylated maltotetrasaccharide (Dp4) was added as internal standard for quantification and is observed at m/z 899.48. **(B)** Intact N-glycans were analyzed in negative ion mode. Expanded view of a subregion of the full MS highlights the presence of *O*-acetylated sialic acid on bi-antennary disialylated and trisialylated N-glycans in channel catfish. **(C)** MS3 fragmentation of the signature ions detected at m/z 1051 corresponding to sialylated tetrasaccharide composed of NeuAc1Hex2HexNAc1 and MS4 fragmentation of the m/z 676 fragment produces fragments corresponding to the neutral loss of 1 hexose and 1 NeuAc, indicating that the epitope is a branched form of the NeuAc(Hex)HexHexNAc tetrasaccharide.

The N-glycans of channel catfish sera were analyzed as their permethylated and native forms. N-glycan profiles of three biological replicates are shown in Supplementary Figure S5E. Representative N-glycan profiles are shown in Figure 4. For permethylated forms of N-glycans, the most abundant ion, observed at m/z 1082.52, corresponds to a bi-antennary N-glycan with a monosaccharide composition of NeuAc2Hex7HexNAc4 and possessing a unique glycan motif composed of NeuAc1Hex2HexNAc1. The most complex N-glycan structure detected was a tetra-antennary glycan detected at m/z 1183.82 (4+) corresponding to a composition of NeuAc3Hex10HexNAc6. We also identified N-glycan at m/z 962.13 (3+)/1431.70 (2+) corresponding to a composition of NeuAc1Hex7HexNAc4 and carrying one non-sialylated form of a glycan epitope composed of Hex2HexNAc1 (Figure 4A). Native, non-permethylated forms of N-glycans were analyzed in negative ion mode and detected the presence of between zero and two *O*-acetyl groups (Figure 4B).

**FIGURE 5:**
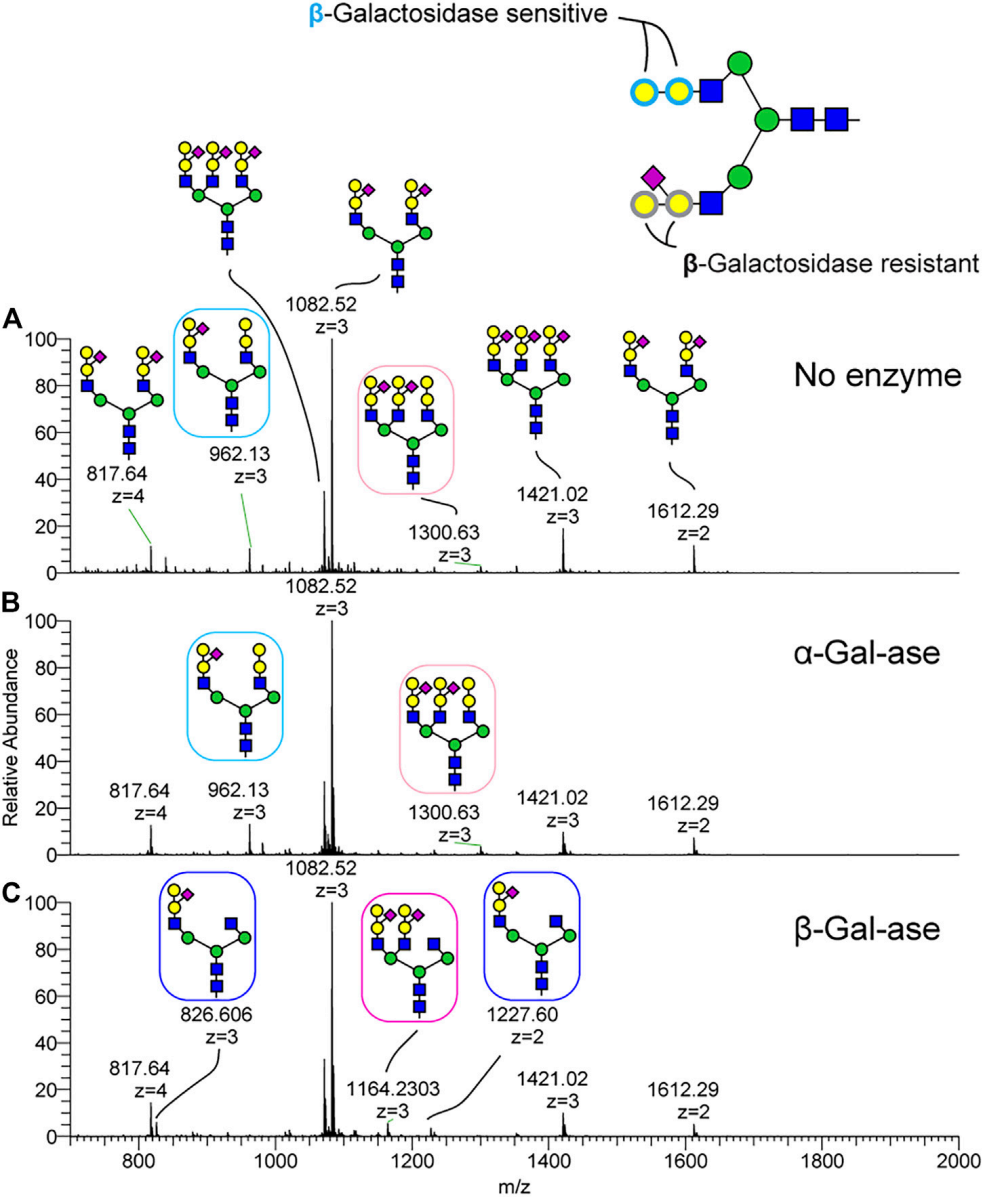
Validation of the channel catfish terminal glycan motif by exoglyosidase treatment. N-Glycopeptides harvested from channel catfish serum were treated with α and β-galactosidases and the N-Glycans were released by PNGaseF and analyzed as their permethylated derivatives by NSI-MS/MS. **(A)** N-glycan profile without enzyme digestion. **(B)** N-glycan profile following α-galactosidase digestion. **(C)** N-glycan profile following β-galactosidase digestion. The glycan motif composed of Hex-Hex-HexNAc trisaccharide (e.g., boxed N-glycan at m/z 962, 3+ and 1300, 3+) is digested to HexNAc by β-galactosidase but is resistant to α-galactosidase. The sialylated trisaccharide was not susceptible to β-galactosidase digestion.

To further characterize the topology of the unique NeuAc1Hex2HexNAc1 motif, sequential MSn analysis was carried out for m/z 1082. MSn fragmentation for this structure is shown in Figure 4C and Supplementary Figure S6. We observed a signature MS2 fragment ion at m/z 1051, which corresponds to a monosaccharide composition of NeuAc1Hex2HexNAc1 (Supplementary Figure S6). MS3 at m/z 1051 yields a neutral loss of sialic acid from the glycan epitope producing a fragment ion at m/z 676 (Figure 4). MS4 for the trisaccharide detected at m/z 676 indicates that sialic acid is attached on the internal Hex of the Hex1-Hex1-HexNAc1 trisaccharide. Thus, the glycan sequence of tetrasaccharide is identified as NeuAc1(Hex1)Hex1HexNAc1. To orthogonally validate the glycan sequence of this tetrasaccharide motif, N-glycans were treated with α- and β-galactosidases (Figure 5). The non-sialylated form of the epitope (Hex2HexNAc1) was sensitive to β-galactosidase, but it was completely resistant to α-galactosidase. The sialylated form of the glycan epitope (NeuAc1(Hex1)Hex1HexNAc1) was resistant to both α- and β-galactosidases, indicating that addition of the branching sialic acid on this trisaccharide inhibits digestion. MS analysis demonstrated that two galactose residues of Hex1Hex1HexNAc1 trisaccharide were completely digested to the HexNAc residue by β-galactosidase (Figure 5), demonstrating the desialylated motif sequence to be GalβGalβHexNAc. The observed ions and their glycan compositions are summarized in Supplementary Table S1E.

## Discussion

**FIGURE 6:**
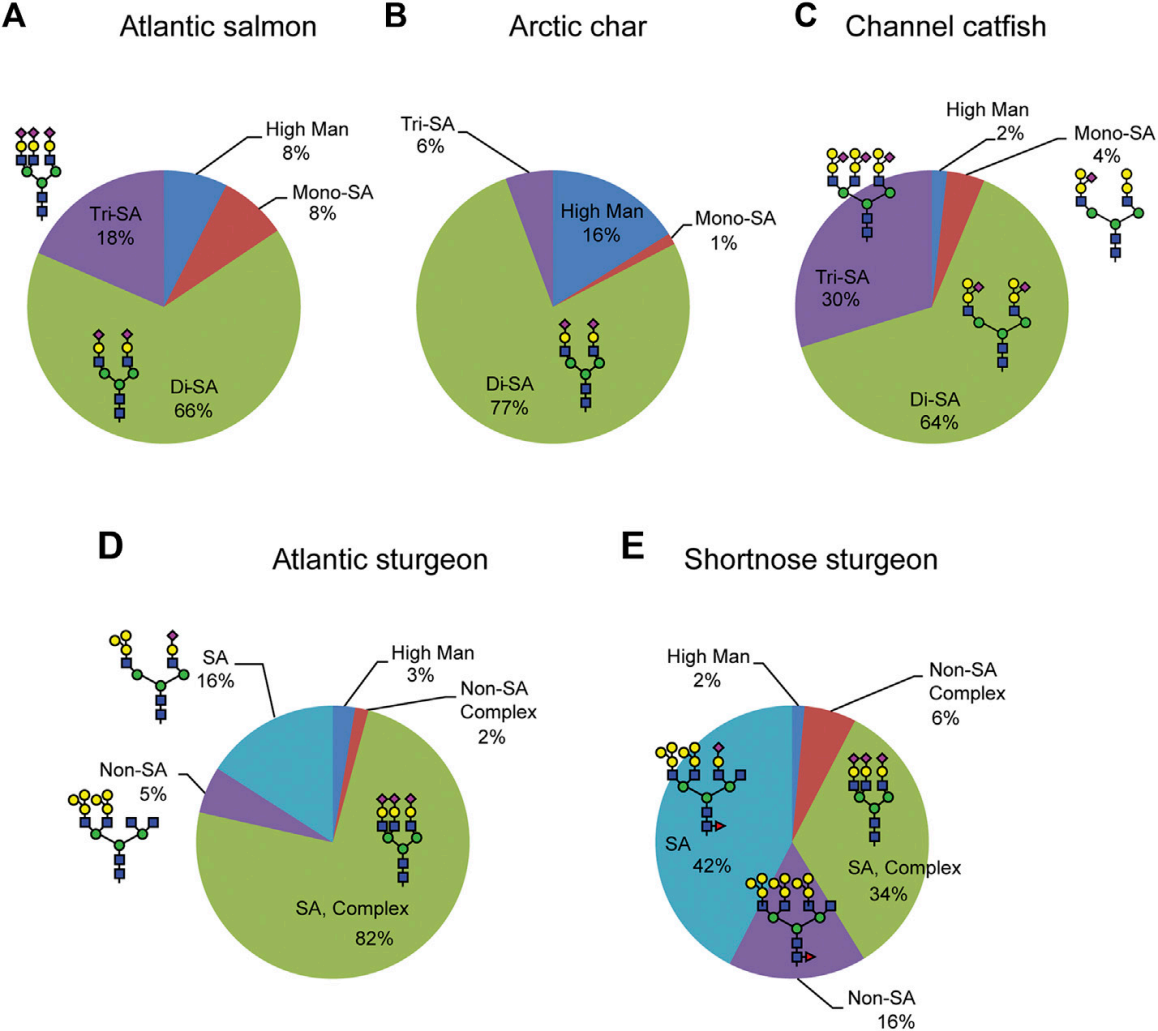
Relative quantification of N-glycan structural features. Glycans were released by PNGaseF from fish sera glycoproteins, permethylated, identified and quantified by NSI-MSn. The prevalence of the major glycan features are expressed as a percent of the total profile of detected glycans.

The serum samples that we subjected to N-glycome anlaysis were harvested from three teleost fish species (Atlantic salmon and Arctic char from family *Salmonidae*, channel catfish from family Ictaluridae), and from two non-teleost, condrostrean fish species (Atlantic and shortnose sturgeon from family Acipenseridae). Here we provide an analysis of the serum N-glycome in five selected fish species, but the glycan-related genes in those species have never been analyzed. Currently transcriptome analysis was only carried out for model fishes such as zebrafish (*Danio rerio*) and Japanese Medaka (*Oryzias latipes*) (Mathavan et al., 2005; Kasahara et al., 2007). Relative quantification of N-glycans from these fish sera demonstrate that different fish species express unique glycan motifs in terms of oligosaccharide sequence, sialylation pattern, the degree of *O*-acetylation of sialic acid, and fucosylation of chitobiose cores (Figure 6). Comparative phylogenetic studies of the glycomes in different organisms are rare, but they generally indicate higher rates of divergence in glycan structures than in protein or DNA (Larkin and Imperiali, 2011; Bhavanandan and Gowda, 2014; Seeberger, 2015; Springer and Gagneux, 2016). For serum glycomics, a major contributor to the whole glycome of most organisms is immunoglobulin (Ig) glycosylation. While protein-specific glycomic analysis (glycoproteomics) remains to be undertaken in the species reported here, we anticipate that a significant fraction of their total serum glycome is contributed by Ig glycosylation; the detection of highly abundant biantennary glycans in these fish is consistent with Ig glycosylation in other species.

**FIGURE 7:**
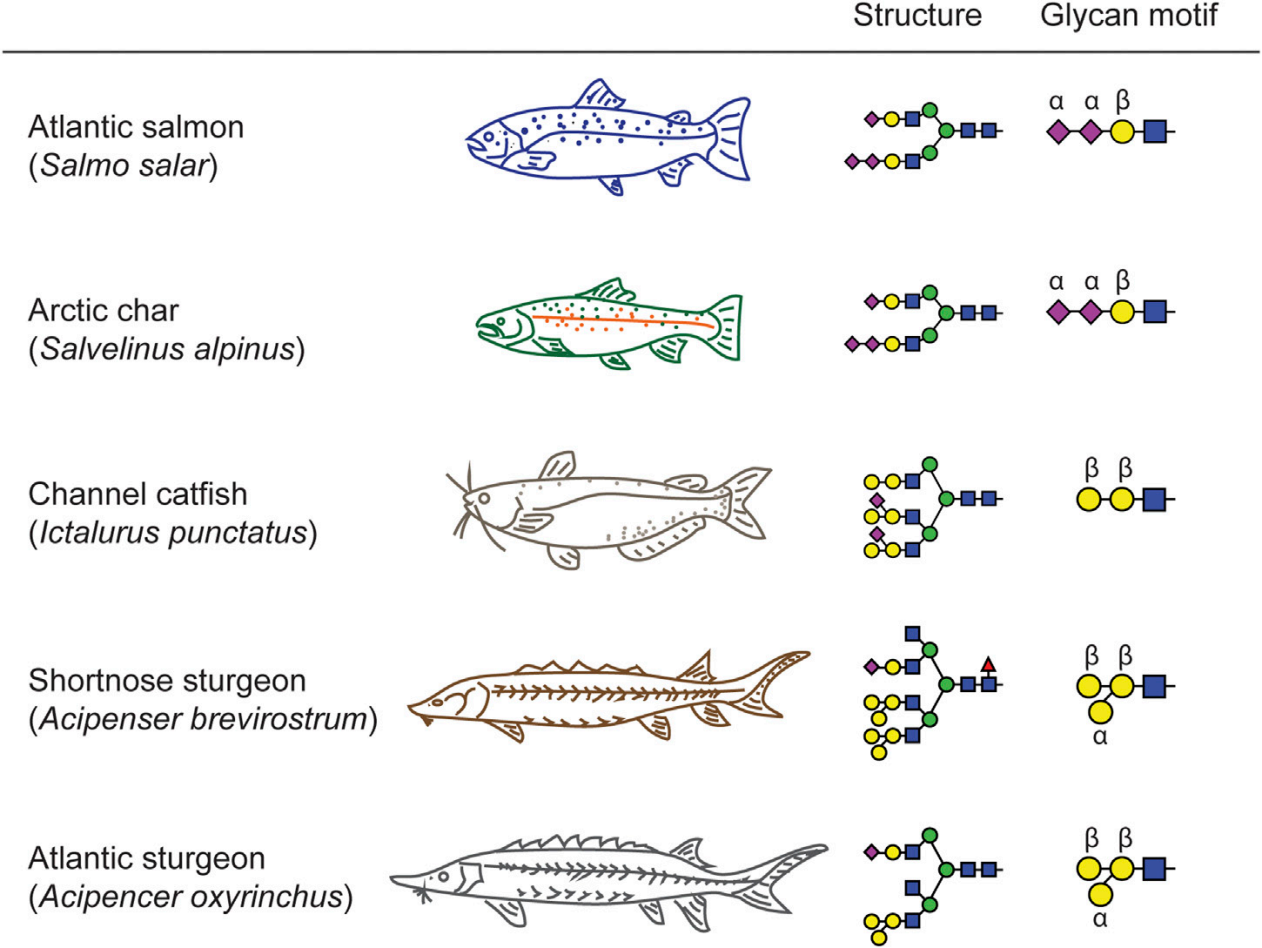
Signature motifs of teleost and chondrostrean serum glycans. The *Salmonidae* species express terminally mono- and di-sialylated glycans (Atlantic Salmon and Arctic Char). The chondrostrean species of sturgeon possess GalβGalβ motifs branched with additional Galα residues. The other teleost analyzed in this study, the channel catfish, also possesses the GalβGalβ motif, but branches it with sialic acid rather than Gal, thereby presenting a hybrid structure that blends elements of the *Salmonidae* and chondrostrean sturgeon motifs.

In general, fish Ig exists in serum as a high molecular weight glycoprotein that mostly shares structure, gene organization, and physicochemical properties with mammalian immunoglobulin M (IgM). For example, there are three major Ig isotypes in salmonid fish, IgM, IgD and IgT (Hikima et al., 2011). The major systemic antibody in teleost fish is IgM, while IgT is specific to mucosal immune responses (Zhang et al., 2010; Tadiso et al., 2011). Differences also exist in the Ig expression patterns of teleost fish. The IgT isotype of rainbow trout occurs as a monomer in blood and a tetramer in mucus, while channel catfish lack the IgT isotype entirely (Bengtén et al., 2002; Liu et al., 2016). Although the protein structure of Ig may be shared across teleost and condrostrean fish species, we have demonstrated that the glycan moieties on serum proteins in these groups of fishes possess common features but also unique motifs (Figure 7). These characteristic glycosylation profiles may contribute to species-specific differences in the regulation of inflammatory and immune responses in fish biology.

**FIGURE 8:**
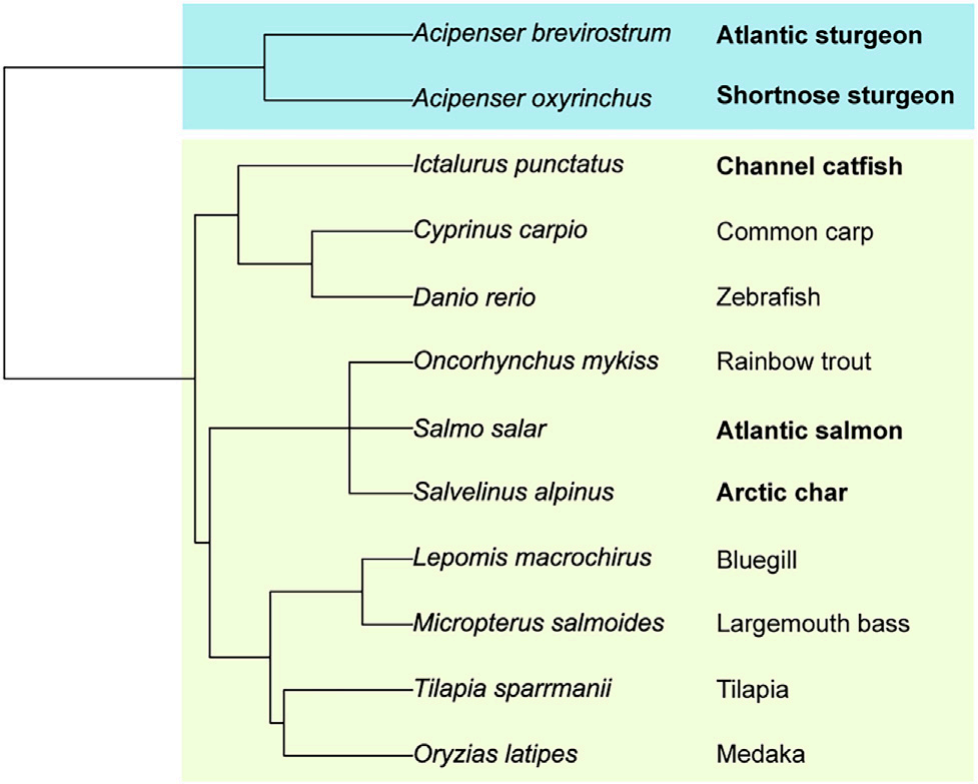
Phylogenetic relationships between teleost and chondrostrean fish species. The evolutionary tree was rooted on selected teleost (boxed in light green) and chondrostrean fishes (boxed in blue). Phylogenetic separations of the species based on genomic similarities are paralleled by glycomic diversity.

We created a fish phylogenetic tree using FishPhyloMaker to define the evolutionary distance relationships between a broad selection of teleost and condrostrean fish species (Figure 8; Nakamura et al., 2021). As expected, the sturgeon family of condrostrean fishes is clearly separated from the teleost fishes. Our glycomics analysis parallels this separation in that the signature glycan-motif of the sturgeon species is significantly different from teleost fish species (Figure 7). Within the three teleost species we analyzed, the Salmonidae motif (terminal sialic acid and di-sialic acid) is distinct from the Ictaluridae (channel catfish) motif (Galβ-Galβ with branching sialic acid), which shares the backbone Galβ-Galβ disaccharide with the sturgeon motif. Thus, these structural features are blended across families to achieve species-specific glycosylation.

It would be of interest to extend our serum glycomic analysis to the coelacanth, an organism more closely related to the terrestrial tetrapods. Genomic analysis has so-far failed to identify IgM-like genes in coelacanth, even though all other major molecules of the immune system were identified (Amemiya et al., 2013). Instead, two IgW genes, homologous to genes found only in lungfish and cartilaginous fish, were identified in coelacanth. The IgW gene has been suggested to have originated in the ancestor of jawed vertebrates, but was then subsequently lost in teleosts and tetrapods. Determining whether coleocanth serum glycosylation segregates with Ig inheritance, i.e., is unique from teleosts, or has evolved in a convergent way would be informative regarding the breadth of glycan diversity that is consistent with fish biology and immune response.

Consistent with previous reports, we detected *O*-acetylated sialic acids in the Salmonidae serum we analyzed. We also detected *O*-acetylated sialic acids in the catfish and sturgeon samples, although at much lower abundance. Sialic acids are involved in host-pathogen interactions in various diseases across many species of animals. *O*-acetylation of sialic has been widely observed in fish skin and on intestinal mucins and contributes to immune responses against microbial infection (Venkatakrishnan et al., 2019). Desialylation of skin and intestinal mucins of Atlantic salmon has been shown to reduce *Aeromonas salmonicida* binding to these glycoproteins. Sialic acid imparts important physicochemical properties to the proteins it modifies, including mucins. Due to their large size, high amount of negative charge, and extreme hydrophilicity, mucins and their sialylated glycans contribute to mucus-based physical and chemical protection of cells. Similarly, sialylation and sialic acid *O*-acetylation of serum glycoproteins likely contributes to immune protection of fish against pathogen invasion.

“Catfish” (order Siluriformes) represent 12% of teleost fish species or 6.3% of all vertebrate species, and several are of enormous economic value to world aquaculture (Liu et al., 2016). Although channel catfish and the two salmonid species investigated are all teleost fish, Siluriform fish emerged about 10 million years before the salmonids (Taylor et al., 2001; Volff, 2005). They are phylogenetically distinct and our glycomic analysis revealed that the structure of their N-glycans distinctly differ. Channel catfish exhibit less *O*-acetylation of sialic acid and express a unique glycan motif composed of Galβ(NeuAc)GalβHexNAc and a nonsialylated form of the GalβGalβHexNAc motif. The trisaccharide motif of GalαGalβGlcNAc has been detected in mammals, including swine and mice but not in humans. However, the anomeric configuration of the mammalian type epitope, GalαGalβGlcNAc, differs from the GalβGalβGlcNAc configuration observed in this study and may represent a glycan motif specific to fish. The sialylated form of this glycan epitope was completely resistant to β-galactosidase, suggesting that the addition of sialic acid to GalβGalβHexNAc produces a branched form of the glycan that could confer resistance to bacterial glycosidases. Alternatively, it remains possible that further structural analysis by orthogonal methods, such as NMR and GC-MS of permethylated alditol acetates prepared from catfish glycans, may reveal subtle structural features that we have not resolved by our current methods. These approaches will become valuable when sufficient glycan can be prepared from biological sources. Interestingly, channel catfish did not express the bi-antennary disialylated and tri-antennary trisialylated N-glycans observed in the salmonds. Surprisingly, we were not able to detect N-glycans carrying NeuGc and KDN in Atlantic salmon or Arctic char sera although they were found in the skin and intestinal mucins of salmon species as their O-glycan components (Jin et al., 2015; Venkatakrishnan et al., 2019; Benktander et al., 2020). NeuGc N-glycans were barely detected on MS2 and were not detectable on the MS1 level.

Sera from the Atlantic sturgeon and shortnose sturgeon was analyzed by N-glycan profiling. The structures of N-glycans in the chondrostrean species seem more complex than those of teleost fish. However, there are some similarities in terms of the structure of their glycan motifs. As described above, channel catfish carry a unique glycan motif consisting of GalβGalβHexNAc, the same motif was detected as a minor glycan component in both sturgeon species, although the sialylated form of the epitope was not detected. Interestingly, the sturgeon N-glycan motif carries an additional hexose on GalβGalβHexNAc, to build a tetrasaccharide glycan motif of Galβ(Galα)GalβHexNAc (Phelps et al., 2003; Nairn et al., 2012; Huai et al., 2016). Although sturgeon do not carry a sialylated form of this epitope, the branched form of this motif may contribute in resistance to bacterial invasion by inhibiting the activity of microbial exoglycosidases. A significant difference was also observed between the two sturgeon species regarding fucosylation of the chitobiose core, which was more abundant (more than 40%) in shortnose sturgeon compared to 2% in Atlantic sturgeon (Figure 6; Supplementary Tables S1C,D). Although there is a much greater diversity of N-glycan structures in sturgeons, N-glycans with NeuGc and KDN were not observed in sturgeon sera analyzed in this study.

The lack of NeuGc and KDN in the sera we studied is of interest since other fish species, notably zebrafish, are rich in these other sialic acids. The evolutionary divergence between teleosts and other fish species has been characterized at genetic and genomic levels. More than 99.8% of ray-finned fishes belong to the teleosts, but bichirs and sturgeons are examples of non-teleost ray-finned fishes. Sequence divergence analysis between paralogs suggests that channel catfish and zebrafish diverged 110–160 million years ago. Species like the Atlantic salmon and rainbow trout emerged 25–100 million years ago by tetraploidization events, while sturgeon and paddlefish emerged 180–200 million years ago (Volff, 2005; Lien et al., 2016). It has also been reported that up to 25% of the salmon genome experienced delayed re-diploidization after the earlier large chromosome rearrangements (Taylor et al., 2001). Therefore, sturgeons are considered as one the earliest diverging fish species, with channel catfish and the Salmonidae following. Since each of these species were devoid of NeuGc and KDN in our serum analysis, the diversification of sialic acid may have begun as recently as 25 million years ago in the teleosts. Alternatively, the lineages that we analyzed may have separately lost the capacity to produce these sialic acids. Targeted genomic analysis will be required to distinguish between these possibilities.

In conclusion, we profiled fish serum N-glycans from five fish species, thereby expanding the N-glycan database among both teleost and chondrostrean fishes. Similar fish serum glycomics studies have only been conducted for grass carp and juvenile Atlantic salmon (Jayo et al., 2012; Su et al., 2018). While all but the most ancient vertebrates possess an antibody producing immune system, the degree of conservation among these systems and the relatedness of antibody glycosylation remains unknown. Nonetheless, viral infection of teleost fish induces a typical antibody response (Ourth, 1982; Ourth and Bachinski, 1987; Guselnikov et al., 2018). Therefore, expanding our knowledge of serum immunoglobulin glycosylation in fish could lead to better understanding of fish immune systems and their responses to pathogens. Over the past decades, comprehensive and systematic investigations have shown that animal species express sets of unique glycan structures that frequently define their distinctiveness and responsiveness to environmental or pathological stress (Plomp et al., 2016; Gudelj et al., 2018; de Haan et al., 2020). Branching, extension, and post-synthetic glycoprotein-glycan modifications (acetylation, methylation, sulfation, phosphorylation, etc.) are highly variable across species and across cell types within any one species. While the conservation of core glycosylation mechanisms indicates the essential nature of this protein modification, the diversity of glycan structural elaboration across species suggests that patterns of glycan motifs may contribute to evolutionary divergence among animal species. By continuing to expand the number of well-characterized animal glycomes, the patterns and mechanisms that sculpt glycomic diversity through evolution will become increasingly apparent.

## Data Availability

The datasets presented in this study have been deposited in the GlycoPost repository under the accession number GPST000210.

## Supplementary Material

Supplementary Figure S1 NSI-MSn fragmentation of permethylated N-glycans with disialo motif harvested from Atlantic salmon. Permethylated N-glycans were analyzed by NSI-MSn. Fragmentation of the molecular ions corresponding to permethylated NeuAc3Hex2HexNAc2M3N2 (m/z = 1066.84, z = 3) or permethylated NeuAc4Hex3HexNAc3M3N2c (m/z = 1336.98, z = 3) demonstrate that both carry a NeuAc-NeuAc disaccharide whose signature ion is detected at m/z = 759. **(A)** N-glycan with composition of NeuAc3Hex2HexNAc2M3N2 yields strong fragment ions at m/z = 942 (3+) and 1401 (2+) which correspond to a loss of the non-reducing terminal NeuAc from each respective structure. **(B)** N-glycan with composition of NeuAc4Hex3HexNAc3M3N2c also yields strong fragment ions at m/z = 1212 (3+) and 1806 (2+) which correspond to a loss of the non-reducing terminal NeuAc from each respective structure. Both structures demonstrated signature ions corresponding to NeuAc-NeuAc-Hex-HexNAc (m/z = 1208) and NeuAc-Hex-HexNAc (m/z = 847), respectively.

Supplementary Figure S2 In-source fragmentation of permethylated N-glycans harvested from shortnose sturgeon. Permethylated N-glycans were analyzed by NSI-MS employing in-source fragmentation to detect fragments consistent with signature structural motifs. In-source fragmentation was set at 100% energy to produce fragment ions efficiently. **(A)** N-glycan profile with ISF energy 0%. **(B)** N-glycan profile with ISF energy 100%. Signature ions derived from NeuAc1Hex1HexNAc1 and Hex3HexNAc1 were observed at m/z 847 and 894 when ISF energy 100% was applied. **(C)** N-glycan profile following α-galactosidase with ISF energy 0%. D) N-glycan profile with α-galactosidase with ISF energy 100%. Signature ions derived from NeuAc1Hex1HexNAc1 and Hex2HexNAc1 were observed at m/z 847 and 690 when ISF energy 100% was applied. The tetrasaccharide composed of Hex3HexNAc1 (m/z 894) is quantitatively shifted to Hex2HexNAc1 (m/z 690) by treatment with α-galactosidase.

Supplementary Figure S3 In-source fragmentation of permethylated N-glycans harvested from Atlantic sturgeon. Permethylated N-glycans were analyzed on NSI-MS employing in-source fragmentation to detect fragments consistent with signature structural motifs. In-source fragmentation was set at 100% energy to produce fragment ions efficiently. **(A)** N-glycan profile with ISF energy 0%. **(B)** N-glycan profile with ISF energy 100%. Signature ions derived from NeuAc1Hex1HexNAc1 and Hex3HexNAc1 were observed at m/z 847 and 894 when ISF energy 100% was applied, respectively. **(C)** N-glycan profile with α-galactosidase with ISF energy 0%. **(D)** N-glycan profile with α-galactosidase with ISF energy 100%. Signature ions derived from NeuAc1Hex1HexNAc1 and Hex2HexNAc1 were observed at m/z 847 and 690 when ISF energy 100% was applied. The tetrasaccharide composed of Hex3HexNAc1 (m/z 894) is quantitatively shifted to Hex2HexNAc1 (m/z 690) by treatment with α-galactosidase.

Supplementary Figure S4 Detection of *O*-acetylated sialic acid on N-glycans of Atlantic sturgeon in negative ion mode. N-Glycans released from serum glycoproteins by PNGaseF were analyzed without permethylation as their native forms. Expanded view of a subregion of the full MS shows evidence for the presence of a single *O*-acetyl moiety on a sialic acid of a bi-antennary disialylated N-glycan.

Supplementary Figure S5 Reproducibility of N-glycan profiles across biological replicates. Serum sampled from three separate individual animals were subjected to N-glycan analysis. Representative full MS profiles are shown for permethylated glycans analyzed in positive mode by NSI-MS of biological replicates of **(A)** Atlantic salmon, **(B)** arctic char, **(C)** Atlantic sturgeon, **(D)** shortnose sturgeon, and **(E)** channel catfish.

Supplementary Figure S6 N-Glycans detected in serum of channel catfish by NSI-MSn. N-Glycans released from fish sera glycoproteins by PNGaseF were analyzed as their permethylated forms. **(A)** Full MS spectra of permethylated N-glycans analyzed in positive ion mode demonstrate that the most abundant N-glycan is a bi-antennary disialylated N-glycan detected at m/z 1082 (3+) and 1612 (2+). **(B)** Fragmentation of the molecular ions corresponding to permethylated NeuAc2Hex4HexNAc2M3N2 (m/z = 1062.30, z = 2) demonstrates the presence of sialylated tetrasaccharide composed of NeuAc1Hex2HexNAc1 detected as a fragment ion at m/z 1051.

Supplementary Table S1 (xls), Summary table describing the details (composition, theoretical mass, detected mass, amount, and relative abundance) of glycans detected in fish serum; **(A)** Atlantic salmon, **(B)** arctic char, **(C)** Atlantic sturgeon, **(D)** shortnose sturgeon, and **(E)** channel catfish.
